# Correspondence regarding Zhong et al., BMC Bioinformatics 2013 Mar 7;14:89

**DOI:** 10.1186/s12859-014-0347-5

**Published:** 2014-11-28

**Authors:** Alexandre Kuhn

**Affiliations:** Microfluidics Systems Biology Lab, Institute of Molecular and Cell Biology, Agency for Science, Technology and Research, Proteos Building, Room #03-04, 61 Biopolis Drive, Singapore, 138673 Singapore

## Abstract

Computational expression deconvolution aims to estimate the contribution of individual cell populations to expression profiles measured in samples of heterogeneous composition. Zhong et al. recently proposed Digital Sorting Algorithm (BMC Bioinformatics 2013 Mar 7;14:89) and showed that they could accurately estimate population-specific expression levels and expression differences between two populations. They compared DSA with Population-Specific Expression Analysis (PSEA), a previous deconvolution method that we developed to detect expression changes occurring within the same population between two conditions (e.g. disease versus non-disease). However, Zhong et al. compared PSEA-derived specific expression levels across different cell populations. Specific expression levels obtained with PSEA cannot be directly compared across different populations as they are on a relative scale. They are accurate as we demonstrate by deconvolving the same dataset used by Zhong et al. and, importantly, allow for comparison of population-specific expression across conditions.

**Electronic supplementary material:**

The online version of this article (doi:10.1186/s12859-014-0347-5) contains supplementary material, which is available to authorized users.

## Main text

Gene expression profiling is often performed on biological samples composed of several different cell populations. Notable examples of important biomedical relevance are tumors, blood or brain samples. Expression profiles obtained from heterogeneous samples can be thought of as mixtures of expression contributed by the individual cell populations. Computational expression deconvolution aims to estimate the expression profiles of individual populations or the fraction of each population in the samples. Deconvolution is thus useful to derive expression profiles of cell populations that cannot be easily isolated (e.g. [1]), to study dynamic changes in the abundance of cell populations (e.g. [2]) and to detect expression changes within specific cell populations (e.g. [3,4]).

Adding to the growing body of literature on the subject, Zhong et al. have recently proposed Digital Sorting Algorithm (DSA) to estimate the expression profiles of individual cell populations in heterogeneous samples [5]. Relying on the use of marker genes (i.e. genes specifically expressed in one of the cell populations and not in the others), their method first estimates the fractions of the various cell populations in each sample and then uses them to estimate the expression profiles of individual cell populations. They tested their method on artificially mixed samples of liver, brain and lung tissues originally provided by Shen-Orr et al. [3]. By comparing with expression profiles measured from pure liver, brain and lung samples, they showed that DSA could accurately estimate expression in the individual populations (Figure one, panels a-d in [5]). They then investigated if DSA-deconvolved expression profiles allowed them to correctly detect differences between specific expression levels in 2 different populations. For instance, they showed that they could sensitively and specifically detect 2-fold expression differences between liver and brain (Figure one, panel e in [5]) or between liver and lung (Figure one, panel f in [5]).

We previously proposed to use marker genes for deconvolution and introduced Population-Specific Expression Analysis (PSEA) [4]. PSEA was developed to detect expression changes within a specific cell population across different conditions (e.g. disease versus non-disease). It thus parallels the standard differential expression analysis used to compare gene expression across 2 conditions based on homogeneous samples. We used PSEA to analyze expression profiles obtained from human brain samples and uncovered novel changes in gene expression in neurons of Huntington disease patients [4]. In a separate study, PSEA allowed us to discover age-related changes that were specific to astroctyes, another important brain cell population [6]. Expression profiles obtained with PSEA, however, are on a relative scale that depends on the choice of marker genes used for deconvolution. Levels of expression in different cell populations thus cannot be directly compared with each other. This feature of PSEA is documented in the main text, Methods section and supplementary material of our original publication [4]. In particular, we stated in the latter (see paragraph “Interpretation of population-specific expression level calculated with PSEA”):

“Specific expressions obtained with PSEA are on a relative scale that depends on the selected marker genes (see Methods). As a consequence, specific expressions cannot be compared across different populations: if a particular gene shows identical specific expressions in neurons and oligodendrocytes for instance (as per PSEA), this does not imply, in general, that it is expressed at the same absolute level in these two populations.”

**Figure 1:**
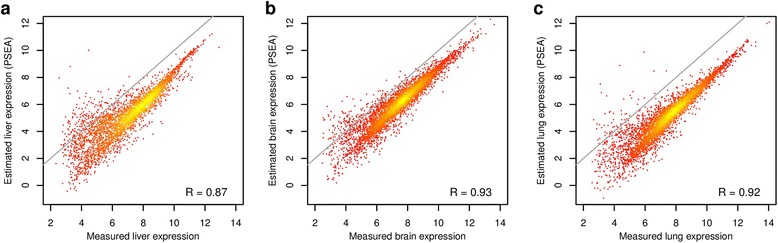
**PSEA-estimated versus measured specific expression. a**: Average expression measured in pure liver samples (x-axis) versus liver expression estimated by PSEA using mixed samples (y-axis). PSEA-estimated expression levels are on a relative scale compared to measured expression and they thus lie parallel to the diagonal (gray line) on this log-log plot. The correlation between estimated and measured expression, however, is high (the correlation coefficient is indicated in the lower right corner). **b**: same as (a) for brain. **c**: same as (a) for lung. The scales of PSEA-estimated expression levels for liver, brain and lung depend on the choice of marker genes used for deconvolution and should not be compared directly.

To compare with DSA, Zhong et al. applied PSEA to deconvolve the liver-brain-lung mixed samples. However, they then proceeded to calculate the differences in PSEA-deconvolved expression profiles between liver and brain (Figure three, panel b in [5]). They observed that the estimated differences in expression between the 2 tissues were biased as compared to true differences and concluded that “the fold change estimated by DSA is more accurate than PSEA”. This conclusion is thus misleading since PSEA was not designed to perform this type of comparison across different populations. As we have shown previously, PSEA provides accurate (but normalized) expression profiles [4,6]. We demonstrate this again with the liver-brain-lung mixed samples used by Zhong et al. For probes that successfully passed deconvolution (7,800 probes using the same criteria as in [4]), specific expression levels showed excellent correlation with measured expression profiles for all 3 populations (see Figure 1 which provides a direct comparison to Figure one, panels b, c, and d in [5]). PSEA-derived expression levels, however, lied parallel to (and not on) the diagonal of these log-log plots as they are scaled compared to measured expression levels. The computer code (R script) used for this analysis is provided as supplementary information (Additional files 1 and 2).

In addition to providing normalized cell population-specific expression levels, PSEA can detect (“fold”) changes within a specific population across 2 or more conditions. This issue is not addressed by DSA as in Zhong et al. Importantly, the scale of population-specific fold changes obtained with PSEA in that case does not depend on the expression range of the marker genes used for deconvolution. Population-specific fold changes can thus be compared between 2 different cell populations: In a comparison of diseased vs. non-diseased brain samples for instance, a 2-fold change in neuronal expression has the same meaning as a 2-fold change in astrocytic expression.

In conclusion, PSEA can accurately deconvolve population-specific expression from heterogeneous samples. As opposed to DSA, estimates are on a relative scale and specific expression levels cannot be directly compared between different cell populations. PSEA, however, was designed to detect differences within the same cell population across 2 or more conditions which is useful for the study of gene expression changes in complex tissues like tumors or brain.

## Response

by Zhandong Liu

Email: zhandong.liu@bcm.edu

Address: Department of Pediatrics Neurology, Computational and Integrative Biomedical Research Center, Jan and Dan Duncan Neurological Research Institute, Baylor College of Medicine, Houston, Texas 77030, USA.

In this correspondence, Kuhn raises two points: (1) PSEA is relative to the cell type markers and should not be used to compute gene expression fold changes between different cell types. (2) PSEA deconvolved expression profiles correlate with the true signals.

Regarding point (1), we agree with the author that PSEA is relative. We have already pointed this out in our original manuscript: “*However, PSEA uses the marker gene information as normalization factors in the gene expression deconvolution analysis. Hence, the estimated gene expression profiles are not the absolute gene expression values, but are relative to the average of the marker genes for each cell type* [1].” Computing fold changes between different cell types is a very important topic in transcriptome analysis. Since our digital sorting algorithm (DSA) does not normalize to different cell type markers directly, DSA is suitable to compute fold changes between different cell types.

Regarding point (2), we noticed that the author used only 13-18% of the total probe sets on the Affymetrix array (Figure 1). Some of the missing gene expressions are due to logarithm transformation of negative values, and the rest are due to p-value filters. One may need to take these probe sets into consideration in order to evaluate the performances of different algorithms.

In summary, I agree that PSEA should be primarily used to compare relative changes of the same cell type across different conditions, which had already been clarified in our original manuscript.

## Acknowledgements

Z.L. is funded by NSF DMS/NIGMS-1263932.

## Reference

1. Zhong Y, Wan Y-W, Pang K, Chow LM, Liu Z: **Digital sorting of complex tissues for cell type-specific gene expression profiles.***BMC Bioinformatics* 2013, **14:**89.

## Additional files

Additional file 1:
**R script to perform PSEA on the liver-brain-lung dataset (GSE19830).**
Additional file 2:
**Marker probe sets used in Zhong et al.**
